# Good practice in medicine and biology: scientific integrity needs global bioethics

**DOI:** 10.1186/s12967-022-03847-0

**Published:** 2023-01-20

**Authors:** Henri-Corto Stoeklé, Achille Ivasilevitch, Christian Hervé

**Affiliations:** 1 Department of Ethics and Scientific Integrity, Foch Hospital, Suresnes, France; 2 Laboratory of Business Law and New Technologies (DANTE) (UR4498), Paris-Saclay University (UVSQ), Montigny-Le-Bretonneux, Paris, France; 3 Medical School, Paris Cité University, Paris, France; 4 Medical School, Versailles Saint-Quentin-en-Yvelines University (UVSQ), Montigny-le-Bretonneux, Paris, France; 5 International Academy of Medical Ethics and Public Health, Paris Cité University, Paris, France; 6 Veterinary Academy of France, Paris, France

**Keywords:** Scientific integrity, Research integrity, Ethics of science, Morality of science, Medicine, Biology, Bioethics, Medical ethics, Research ethics, Global bioethics

## Abstract

We consider *scientific integrity* to constitute a new theory of *morality of science*, in a very specific deontological sense. Indeed, at least in practice, *scientific integrity* extends beyond scientific concerns, seeking to develop specific moral duties and/or procedures based on general moral values and/or standards, leading to common moral frameworks for usual scientific practices. This is, of course, necessary. Contemporary history has shown us only too well that usual scientific practices need common moral frameworks, especially in *medicine* and *biology*. However, like scientific practices, and medical and biological practices in particular, the persistence of certain moral values and/or standards and the priority attributed to them, can change significantly, due to changes in society, people, the times and/or environments, and they may be under strong tension. We therefore believe that a new theory of *ethics of science*, in a very specific teleological sense, may be required in this case, particularly in *medicine* and *biology*, in addition to *scientific integrity*. This ethical theory, through research, professionals and structures in *ethics of science* also called *medical ethics*, *research ethics* or *bioethics* in the fields of *medicine* and *biology*, should seek to identify and find specific ethical solutions to these tensions, applicable at a particular place and time, based on common ethical purposes and/or consequences. As a result, these specific ethical solutions may, or may not, lead to an evolution of common moral frameworks, which may, or may not, be developed on the basis of *scientific integrity.* In the fields of *medicine* and *biology,* this ethical theory is closely related to another theory, *global bioethics,* but with a number of new conceptual and methodological developments.

## Background

*Scientific integrity* is a concept highly relevant to contemporary *medicine* and *biology*. The completely inhumane experiments performed by Nazi doctors during the Second World War alongside their extermination of several million Jews, gypsies, homosexuals, disabled individuals and political opponents led to a veritable awakening of conscience concerning the limits to be imposed on scientific practices. Major international declarations were proclaimed, beginning with the Nuremberg Code after the trial of Nazi doctors just after the war. The Nuremberg code was followed, in 1948, by the declaration of the United Nations, then the Declaration of Helsinki in 1964, the Declaration of Oviedo in 1997 and, finally, that of the United Nations Educational, Scientific and Cultural Organization in 2005. France has had bioethics laws in place since 1994, and many other organizations, including the Council on International Organizations of Medical Sciences have also made similar declarations since 1949. Informed consent (e.g. explicit consent, etc.) and ethics committees (e.g. national ethics committees, etc.) have clearly emerged from all these declarations as two absolute legal and moral rules in *healthcare* and *biomedical research*. However, despite attempts to enforce these rules in hospitals and research institutes in many countries, it has proved impossible to punish all bad scientific practices, especially scientific fraud. *Scientific integrity* thus remains highly relevant to contemporary *medicine* and *biology*, and has been the subject of much discussion [1]. Nevertheless, theoretical and practical issues remain unresolved and merit further clarification and proposals, in our view.

## Theory versus practice

*Scientific integrity* is becoming increasingly important within the scientific community. The Dutch biologist Elisabeth Bik is one of the best-known contributors to the field in *medicine* and *biology* [2]. Bik founded the “Science Integrity Digest”, a blog entirely dedicated to *scientific integrity* and analyses of cases of real or supposed misconduct. She is also a regular participant on the computer platforms “Retraction Watch”, and “Pubpeer", which list and comment on scientific articles that have been retracted (for Retraction Watch) and identify the failings of publications in terms of *scientific integrity* (Pubpeer). Bik may have been a pioneer in this field, but many other individuals from the academic and institutional world have also contributed to its development [3]. S*cientific integrity* came to the fore in the United States in 1989, with the creation of the Office of Scientific Integrity, which was merged with the Office of Research Integrity in 1992. At the start of this century, various international declarations relating specifically to *scientific integrity* were published, including the Singapore Statement on Research Integrity in 2010, and the European Code of Conduct for Research Integrity in 2011. These declarations relate to all branches of science, including *medicine* and *biology*.

In our home country, France, the concept of *scientific integrity* first emerged in 1999, with the creation by the French National Institute for Health and Medical Research of a delegation for scientific integrity. This delegation related specifically to the fields of *medicine* and *biology*. Nowadays, most French universities and the French National Scientific Research Center, through its ethics committee, pay considerable attention to this issue, in *medicine*, *biology* and beyond. The Corvol Report, entitled “Assessment and proposals for the implementation of a national scientific integrity charter” is one of the latest French national and institutional elements to be added in the construction of *scientific integrity* in France, to establish a common moral framework for all scientific practices, including medical and biological practices. This report proposed relatively concrete and relevant solutions for eradicating scientific fraud, which may take various forms, such as the fabrication or falsification of results, plagiarism, questionable research practices (QRPs), conflicts of interest and issues relating to authorship.

Some philosophers differentiate between *scientific integrity* and *research integrity*, probably for etymological reasons *(*i.e. *science* vs. *research)*, but, in some cases, they may wish to differentiate between strictly scientific concerns (i.e. true vs. false = *scientific integrity*) and moral concerns (i.e. good vs. bad = *research integrity*) [4]. However, these theoretical distinctions, made by philosophers, may be much less clear, in practice, to physicians, biologists, and other scientists [5]. In practice, the terms “*scientific integrity”* and “*research integrity”* may be interchangeable to a certain extent in *medicine*, *biology* and other fields of science [6]. Moreover, in France, *scientific integrity* and *research integrity* are grouped together under the single term “*intégrité scientifique*”, which translates as “*scientific integrity*”, in both practice and theory. Furthermore, the Corvol Report clearly stated that *scientific integrity* “should be based on universal moral principles, such as the notions that it is bad to lie or to steal”.

Let us assume, for the sake of argument, that *scientific integrity* and *research integrity* are synonymous, at least in practice, and based as much on *science* as on *morality*. There remains another major theoretical and practical issue that is even more important: *morality,* like *science,* can change significantly over time and may differ between places, mostly due to societies, people, times and/or environment [7]. Even in Western societies, different countries, such as the United States and France, for example, have different views of the roles of public and private funding, with very different relationships between the state and markets. Private funding is favored in the United States, whereas public funding is favored in France, in many areas, including *medicine* and *biology*, and for biotechnologies in particular (e.g. genetic testing, etc.) [8]. Of course, scientific fraud, such as the fabrication and falsification of results, or plagiarism, is condemned both morally and legally in similar ways everywhere. However, the situation is more complex when it comes to conflicts of interest, precisely because the relationship between the state and markets, and, thus, the proportions of private or public funding, differ radically between countries, and no particular approach can be considered morally superior to another. We therefore believe that it is important to take differences between societies, people, times and/or environments into account more effectively in scientific practices, especially in *medicine* and *biology*, without falling into extreme relativism. A very specific theoretical and practical distinction between *ethics* and *morality* would be helpful in this respect.

## Ethics versus morality

*Ethics* has a much longer history than *scientific integrity*, dating back to the Classical era. Nowadays, we can distinguish *normative ethics* (i.e. a moral judgment of an action or attitude according to an ethical theory) from *applied ethics* (i.e. a practical application of *normative ethics* to a particular field, such as *medicine* and *biology*) and *meta-ethics* (i.e. a conceptual analysis of *normative ethics*) [9]. There are also at least two large families of *normative ethics*. The first is *deontological ethics*, which judges the morality of an action or attitude as a function of its conformity to specific moral duties and/or procedures, as in *Kantism* (i.e. an ethical theory), where specific moral duties are categorical imperatives. The second is *teleological ethics*, which judges the morality of an action or attitude according to common ethical purposes and/or consequences, known as *Consequentialism*, as in *Utilitarianism* (i.e. another ethical theory), in which the ethical consequences most frequently considered are the amount of pleasure or suffering.

But is there a difference between *ethics* and *morality*? Again, in Western societies, from Classical times right up to the Middle Ages, the difference between these two terms was purely etymological (i.e. Greek vs. Latin = *ethics* vs. *morality*). It was not until the modern epoch, and the contemporary period in particular, that the meanings of these two words and the semantic distinction between them gradually changed, leading to their definitive adoption by certain contemporary philosophers, such as the French philosopher Paul Ricoeur, for whom *ethics* relates to questioning and an openness of spirit, whereas *morality* relates to a closed system of standards. The work of the Canadian anthropologist Raymond Massé runs along the same lines. In practice, *ethics* and *morality* might be distinguished as follows: *morality* conceptualizes and applies general moral values and standards, whereas *ethics* calls them into question [10].

In our view, we could go further. We could say that *morality*, in addition to conceptualizing them, asks “how” to apply these general moral values (i.e. good attitudes = *to be*) and/or standards (i.e. good actions = *to do*), or, “how” *to be* this and/or *to do* that in the face of usual social practices (i.e. real attitudes = *what is*/real actions = *what is done*). It develops specific moral duties and/or procedures in response, thereby generating common moral frameworks (Table 1a). By contrast, *ethics* asks “why” certain moral values and/or standards should be respected in the face of new social practices, “why” is it important *to be* this and/or *to do* that. Its decisions are based on common ethical purposes and/or consequences (Table 1b). *Morality* confers common moral frameworks on social practices, whereas *ethics* considers these frameworks when tensions develop between new social practices and certain moral values and/or standards, generating ethical issues, for which specific ethical solutions are required. In the end, these specific ethical solutions may, or may not, lead to a change in the common moral frameworks. In this way, *deontological ethics* has clearly been transformed into *morality* (Table 1a), and *teleological ethics* into *ethics* (Table 1b), because *morality* focuses on specific moral duties and/or procedures, whereas *ethics* relates to common ethical purposes and/or consequences.

**Table 1:**
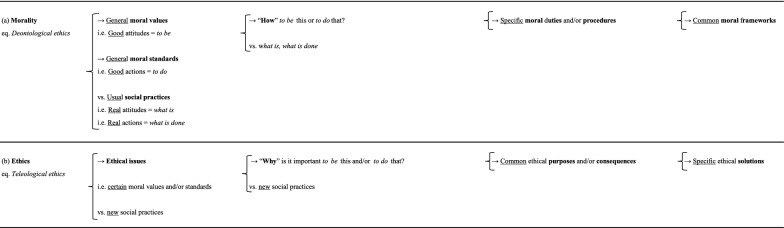
**a** *Morality* (eq. *deontological ethics*); **b** *Ethics* (eq. *teleological ethics*)

Thus, *scientific integrity* corresponds to a new theory of *morality of science*, in a very specific deontological sense (Table 2a). At least in practice,  it extends beyond purely scientific concerns, seeking to develop specific moral duties (e.g. reliability, honesty, respect, accountability, etc.) and/or procedures (e.g. enforcement, punishment, etc.) based on general moral values (e.g. *to be free*, *to be fair*, etc.) and/or standards (e.g. *not to lie*, *not to steal*, etc.), leading to common moral frameworks (e.g. Singapore Statement on Research Integrity, European Code of Conduct For Research integrity, Corvol Report, etc.) for usual scientific practices (e.g. *experimentation*, *observation*, *calculation*, etc.). This is, of course, necessary. Usual scientific practices need common moral frameworks, especially in *medicine* and *biology*, as we have learnt from contemporary history. However, as pointed out above, the meaning, priority or the very existence of certain moral values and standards, just like scientific practices (e.g. *COVID-19 vaccination*, etc.) can change significantly over space and/or time, and be strongly in tension (i.e. ethical issue), due to changes in societies, people, times and/or environments. We, therefore, believe that a new theory of *ethics of science*, in a very specific teleological sense, may be required, particularly in *medicine* and *biology*, in addition to *scientific integrity* (Table 2b).

**Table 2:**
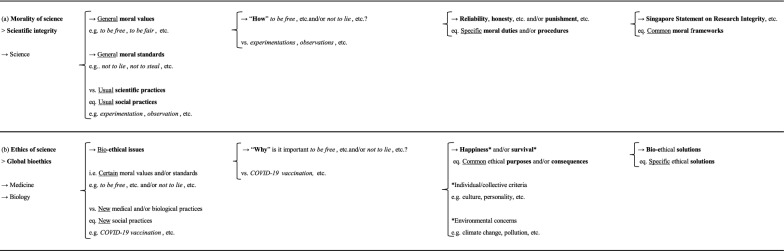
**a** *Morality of science* (eq. *scientific integrity*); **b** *Ethics of science* (eq. )

This theory would seek to identify ethical issues and to find specific ethical solutions to these issues (i.e. these tensions between certain moral values and/or standards and new scientific practices, particularly medical and/or biological practices), in a given spatial and/or temporal context (i.e. societies, people, times and/or environments), based exclusively on common ethical purposes and/or consequences, while taking into account individual and collective criteria (e.g. culture, personality, etc.). As a result, these specific ethical solutions could potentially lead to the evolution of common moral frameworks that could be developed on through *scientific integrity*, on a case-by-case basis, at national and/or international level. In practice, this would involve research (e.g. empirical research, interdisciplinary study, etc.), teachings (e.g. masters, PhD, etc.), structures (e.g. ethics committee, academic department, etc.) and professionals (e.g. bioethicist, full professor, etc.) who should also be scientists, such as physicians or biologists, specializing in *ethics* (e.g. academic degree, scientific publication, etc.) in * ethics of science*.

## Perspectives

At least in *medicine* and *biology,* this ethical theory is closely related to another theory, *global bioethics,* but with several new conceptual and methodological developments [11]. We believe that, within *medicine* and *biology*, these common ethical purposes and/or consequences relate principally to improving the happiness and/or survival of people and/or societies, taking into account different individual and/or collective criteria of happiness and/or survival (e.g. culture, personality, etc.), and environmental concerns (e.g. biodiversity loss, global warming, etc.) (Table 2b). In this way, through a greater pragmatism and pluralism at the conceptual level than for the initial theory, *global bioethics* may be better adapted than other ethical theories (e.g. *principlism*) to the considerable cultural diversity and real needs of humanity, both material (e.g. food, health, etc.) and non-material (e.g. love, spirituality, etc.). It should, therefore, also be better equipped to address (bio-)ethical issues. At the methodological level, interdisciplinary studies (i.e. combining *life sciences*, *human sciences*, etc.), empirical research (i.e. qualitative research, quantitative research, etc.) and conceptual analyses (i.e. *meta-ethics*, *descriptive ethics*, *comparative ethics*, etc.) should be favored. In practice, at Foch Hospital, in France, we made use of this ethical theory during the COVID-19 pandemic, to study different (bio-)ethical issues in *oncology* [12], especially the policy concerning anti-COVID-19 vaccination for cancer patients [13]. We are working on the further conceptual and methodological development of this ethical theory in our department, with the aim, in particular, of differentiating between the study of macro-(bio-)ethical issues (eq. issues at large scales = societies, countries, etc.), and micro-(bio-)ethical issues (eq. issues at local scales = individuals, hospitals, etc.) [14],

Theoretically, *global bioethics* could be developed along more than one route to ensure good practice in *medicine* and *biology* without ever justifying the worst types of behavior (e.g. murder, torture, etc.), even in a multicultural world. However, in practice, even new declarations or organizations in *ethics of science medical ethics*, *research ethics*, *bioethics*, etc. would not be sufficient to achieve this end. More training in *global bioethics* is required, within biology faculties and medical schools, right from the start of university education, or even before, together with more research programs and scientific publications, with the creation of more teams, laboratories, departments, and professional positions in *ethics of science*, in academic institutions, research institutes and hospitals, to put this ethical theory into practice more widely. *Medicine* and *biology* will undoubtedly continue to evolve both scientifically and morally. If we consider animal experimentation as an example, it seems likely that this this practice will one day be banned or that the specific moral duties and/or procedures that already apply to human experimentation will be extended to animal experimentation [15]. However, we believe that only collective and academic (bio-)ethical reflections will make it possible to move beyond individual and militant moral convictions.

Furthermore, in the exponential technological progress occurring in *medicine* and *biology* will also necessitate major educational actions, beyond the training of physicians and biologists. Improvements in patient education and literacy would also be required. At the individual level, this would improve the identification and evaluation of the dangers and risks of these technologies, and at societal level, it would improve their use, both by patients, and by physicians and biologists. We see dynamic consent as a pertinent way of improving patient education and literacy [16]. Modern healthcare administrators and executives in hospitals and research institutes would have a key role to play in this process. However, the recent shift towards *global health* means that patient education and literacy are not the only key ethical issues here [17]. There are also social and/or economic disparities between people and/or societies in terms of access to these technologies. An absence of these technologies in some countries or societies can lead to medical tourism, further deepening these disparities. Moreover, the even more recent shift towards the concept of *one health* means that the spatial and temporal impacts of technologies on ecological (e.g. biodiversity loss, etc.) and environmental (e.g. global warming, etc.) aspects must be also integrated into what is becoming a very complex ethical reflection [18]. *Global bioethics*, with appropriate research methodologies (e.g. action research, systemic modeling, etc.), may prove extremely useful in this context.

## Data Availability

Not applicable.

## Abbreviations

COVID-19: Coronavirus disease 2019
QRPs: Questionable research practices
