# Correction: Good practice in medicine and biology: scientific integrity needs global bioethics

**DOI:** 10.1186/s12967-023-03981-3

**Published:** 2023-03-07

**Authors:** Henri-Corto Stoeklé, Achille Ivasilevitch, Christian Hervé

**Affiliations:** 1 Department of Ethics and Scientific Integrity, Foch Hospital, Suresnes, France; 2 Laboratory of Business Law and New Technologies (DANTE) (UR4498), Paris-Saclay University (UVSQ), Montigny-Le-Bretonneux, Paris, France; 3 Medical School, Paris Cité University, Paris, France; 4 Medical School, Versailles Saint-Quentin-en-Yvelines University (UVSQ), Montigny-Le-Bretonneux, Paris, France; 5 International Academy of Medical Ethics and Public Health, Paris Cité University, Paris, France; 6 Veterinary Academy of France, Paris, France

**Correction: Journal of Translational Medicine (2023) 21:37** 10.1186/s12967-022-03847-0

Following publication of the original article [1], we have been notified that Table 2 legend was incorrectly published. The correct legend of Table 2 should be as per below:

- Table 2 a Morality of science (eq. scientific integrity); b Ethics of science (eq. global bioethics).
